# Screening biomarkers for Sjogren’s Syndrome by computer analysis and evaluating the expression correlations with the levels of immune cells

**DOI:** 10.3389/fimmu.2023.1023248

**Published:** 2023-06-13

**Authors:** Yafang Zhong, Wei Zhang, Dongzhou Liu, Zhipeng Zeng, Shengyou Liao, Wanxia Cai, Jiayi Liu, Lian Li, Xiaoping Hong, Donge Tang, Yong Dai

**Affiliations:** ^1^ Clinical Medical Research Center, Guangdong Provincial Engineering Research Center of Autoimmune Disease Precision Medicine, Shenzhen Engineering Research Center of Autoimmune Disease, The Second Clinical Medical College of Jinan University, Shenzhen People’s Hospital, Shenzhen, China; ^2^ South China Hospital, Health Science Center, Shenzhen University, Shenzhen, China; ^3^ Innovative Markers Department, Fapon Biotech Inc., Dongguan, China; ^4^ Department of Rheumatology and Immunology, Shenzhen People’s Hospital, The Second Clinical Medical College, Jinan University, The First Affiliated Hospital, Southern University of Science and Technology, Shenzhen, China

**Keywords:** Sjogren’s Syndrome, machine learning, potential biomarker, immune cell disturbance, CIBERSORT

## Abstract

**Background:**

Sjögren’s syndrome (SS) is a systemic autoimmune disease that affects about 0.04-0.1% of the general population. SS diagnosis depends on symptoms, clinical signs, autoimmune serology, and even invasive histopathological examination. This study explored biomarkers for SS diagnosis.

**Methods:**

We downloaded three datasets of SS patients’ and healthy pepole’s whole blood (GSE51092, GSE66795, and GSE140161) from the Gene Expression Omnibus (GEO) database. We used machine learning algorithm to mine possible diagnostic biomarkers for SS patients. Additionally, we assessed the biomarkers’ diagnostic value using the receiver operating characteristic (ROC) curve. Moreover, we confirmed the expression of the biomarkers through the reverse transcription quantitative polymerase chain reaction (RT-qPCR) using our own Chinese cohort. Eventually, the proportions of 22 immune cells in SS patients were calculated by CIBERSORT, and connections between the expression of the biomarkers and immune cell ratios were studied.

**Results:**

We obtained 43 DEGs that were mainly involved in immune-related pathways. Next, 11 candidate biomarkers were selected and validated by the validation cohort data set. Besides, the area under curves (AUC) of XAF1, STAT1, IFI27, HES4, TTC21A, and OTOF in the discovery and validation datasets were 0.903 and 0.877, respectively. Subsequently, eight genes, including HES4, IFI27, LY6E, OTOF, STAT1, TTC21A, XAF1, and ZCCHC2, were selected as prospective biomarkers and verified by RT-qPCR. Finally, we revealed the most relevant immune cells with the expression of HES4, IFI27, LY6E, OTOF, TTC21A, XAF1, and ZCCHC2.

**Conclusion:**

In this paper, we identified seven key biomarkers that have potential value for diagnosing Chinese SS patients.

## 1 Introduction

Sjögren’s syndrome (SS) is a systemic autoimmune disease that causes inflammation in exocrine glands, such as salivary and lacrimal glands (1, 2). It may also induce fatigue, musculoskeletal discomfort, disturbance of liver, lung, kidney, nervous system, and lymphoma (3, 4). Studies imply that genetic and environmental variables may be important, even if the pathophysiology of SS is yet unknown (5). The prevalence of SS in different countries is 0.03%-5%, and in China, the prevalence of SS is about 0.33%-0.77% (6, 7). The standard mortality ratio (SMR) of patients with SS ranged from 1.61 to 4.66 in reports from other countries (8–10), and in a previous report, the SMR of patients with SS in China was 3.63 (11). According to the 2016 American College of Rheumatology - European League Against Rheumatism (ACR-EULAR) classification criteria, patients who satisfy the criteria may undergo an invasive procedure—labial gland biopsy (12), which is an arduous and time-consuming invasive examination. In order to reduce the patients’ pain, it is necessary to search for novel non-invasive biomarkers for SS. Taking into account ethnic heterogeneity (13), we would validate the diagnostic value of biomarkers in the Chinese population.

Currently, the diagnosis of SS is a combination of symptoms, clinical signs, histopathology, and autoimmune serology (14, 15). Common diagnostic markers of SS include anti-Ro/SSA and anti-La/SSB antibodies, antinuclear antibodies, and rheumatoid factor, etc. (16, 17). Previous studies have provided a number of new putative serum, salivary and histological biomarkers, such as CXCL13, cathepsin S, IL-4, IL-5, and some type-I and type-II IFN-inducible genes (16, 17). Nevertheless, there is no single clinical, laboratory, pathological, or radiological characteristic that can be considered the “gold standard” for diagnosing SS (18). Researchers are still looking for new disease biomarkers in order to develop simpler, faster methods of diagnosing SS.

As is well known, the immunopathogenesis of SS involves the activation of T and B lymphocytes (19, 20). Many studies reported that dendritic cells, T-helper cells, natural killer (NK) cells showed changes during the development of SS (18, 19, 21). Finding disordered cell subsets associated with pathogenesis can help us better understand the pathogenesis of SS and develop an appropriate therapeutic strategy.

**Figure 1:**
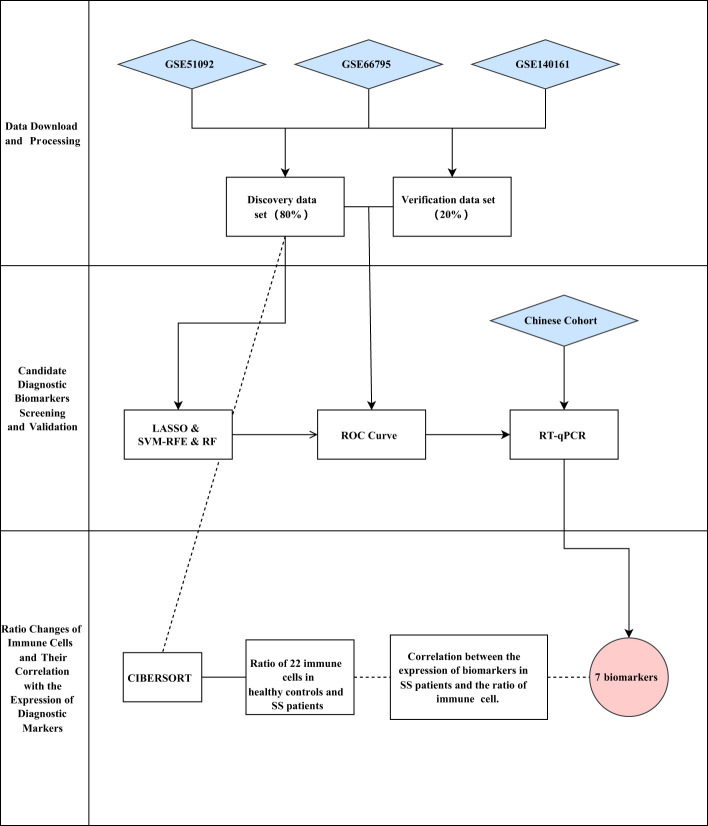
Workflow of the study.

Herein, we downloaded three expression matrix files of SS patients’ and healthy people’s blood samples from the Gene Expression Omnibus (GEO) database. Then, we merged the three expression matrix files into one metadata, and took about 80% of the samples as the discovery cohort (n_SS_ = 522, n_normal_ = 46). Next, we identified the differentially expressed genes (DEGs) between SS and controls. Subsequently, we screened the diagnostic biomarkers of SS through three machine learning algorithms. Following that, we validated the expression of the identified diagnostic biomarkers by using the validation data (about 20% of the metadata cohort, n_SS_ = 150, n_normal_ = 15), and investigated the logistic regression model by the receiver operating characteristic (ROC) curve. Beyond that, we used our own Chinese cohort (n_SS_ = 14, n_normal_ = 10) to validate the expression of the candidate biomarkers. Furthermore, we applied the algorithm of CIBERSORT to calculate the ratio of 22 immune cells in blood samples of SS patients and healthy people. Finally, we investigated the connection between the expression of the identified biomarkers and the ratios of immune cells in blood samples of SS patients. A workflow chart summarizing our work is shown in **Figure 1**.

## 2 Materials and methods

### 2.1 GEO datasets download and data processing

Three expression matrix files (GSE51092, GSE66795, and GSE140161) of SS blood samples were obtained from GEO database. The GSE51092 data set contained 190 SS and 32 healthy controls, the GSE66795 data set contained 131 SS and 29 healthy controls, and the GSE140161 data set contained 351 SS. After batch corrections using the R package “SVA”, the three files GSE51092, GSE66795, and GSE140161 were performed principal component analysis (PCA), which contained the SS patients and healthy controls. Then, the three files were combined into a metadata cohort (n_SS_ = 672, n_normal_ = 61), in which about 80% of the samples were used as the discovery data set (n_SS_ = 522, n_normal_ = 46) and the rest as the verification data set (n_SS_ = 150, n_normal_ = 15).

### 2.2 Identification and functional enrichment analysis of DEGs

The DEGs were identified from the discovery data set using the R package “limma”, and displayed with a volcano plot. The heat map was drawn using the R package to show the expression of DEGs. Then, gene ontology (GO) and Kyoto Encyclopedia of Genes and Genomes (KEGG) pathway were conducted to analyze the functions of the DEGs in the DAVID database online. The gene set enrichment analysis (GSEA) was used to pick the most important pathway between SS and controls. The gene set “c2.cp.kegg.v7.0.symbols.gmt” was selected as the reference gene set from the Molecular Signatures Database (MSigDB).

### 2.3 Screening of the candidate diagnostic biomarkers

The least absolute shrinkage and selection operator (LASSO) logistic regression, support vector machine-recursive feature elimination (SVM-RFE), and random forest (RF) were used to screen the potential SS diagnostic markers. To prevent overfitting the data set, the LASSO analysis was performed using the R package “glmnet”, and based on support vector machine, the SVM-RFE was used to find the optimum variables. The candidate diagnostic markers selected by the three algorithms were overlapped, and the resulting intersection genes were further studied. Then, the candidate diagnostic biomarkers’ expressions were verified in the verification data set.

### 2.4 The ROC curve for diagnostic capability of biomarkers in SS

To investigate the diagnostic capability of the candidate markers, ROC curves were plotted according to the discovery data set and the validation data set. Combined ROC curves were also produced to assess the combined diagnostic utility of candidate markers.

### 2.5 Sample collection and reverse transcription quantitative polymerase chain reaction validation

In total, the whole blood of 14 SS samples and 10 control samples was obtained from Shenzhen People’s Hospital. All participants volunteered to enter this research. Patients who were included in the study were confirmed SS patients according to ACR-EULAR classification criteria, and the clinical manifestations of them were sorted out in **Table 1**. The age of the patients ranged from 29 to 66, and the control group ranged from 26 to 37. The anti-SSA antibodies were positive in 92.9% of the patients, and the anti-SSB antibodies were positive in 57.1% of the patients. 78.6% and 85.7% of the patients had xerostomia and xerophthalmia, respectively. Five patients met the criteria of Schirer’s test ≤ 5mm/5 min in at least 1 eye. This study was approved by the Ethics Committee of Shenzhen People’s Hospital (LL-KY-2019514).

**Table 1:** The clinical manifestations of the 14 SS patients and 10 healthy people in our study.

	SS (n=14)	Healthy people (n=10)
**Sex, Female(percentage)**	14 (100%)	9 (90%)
**Age (year), median (range)**	46 (29-66)	31 (26-37)
**Xerostomia (percentage)**	11 (78.6%)	NA
**Xerophthalmia (percentage)**	12 (85.7%)	NA
**Anti-SSA (percentage)**	13 (92.9%)	NA
**Anti-SSB (percentage)**	8 (57.1%)	NA
**MSG biopsy (percentage)**	3 (21.4%)	NA
**Schirmer’s test ≤ 5mm/5 min in at least 1 eye (percentage)**	5 (35.7%)	NA
**IgG (g/L), median (range)**	18 (12.36-26.06)	NA
**RF (IU/ml), median (range)**	39 (8.4-122.1)	NA

SS, Sjögren’s syndrome; MSG, Minor salivary gland; RF, Rheumatoid factor; NA, not applicable.

When the whole blood samples were obtained, the peripheral blood mononuclear cells (PBMCs) were collected after being diluted with an equal volume of phosphate-buffered saline (PBS) and Ficoll and centrifuged at 2000 rpm for 20 min, and the red blood cell was lysed with red blood cell lysis buffer (Beyotime, C3702). Finally, the PBMCs were mixed with 1mL Trizol (Beyotime, R0016) and stored at -80 °C.

According to the manufacturer’s instructions, total RNA was extracted from PBMCs. The transScript all-in-one first-strand cDNA synthesis superMix for qPCR (One-step gDNA removal) kit (TransGen Biotech, AT341-02) was utilized for the reverse transcription of mRNA. Following that, the RT-qPCR tests were undertaken by the PerfectStart Green qPCR SuperMix kit (TransGen Biotech, AQ601-02). The list of primers is showed in **Table 2**. All of the primers used in our study were synthesized by the Sangon Biotech Company. The housekeeping gene GAPDH was used as an internal reference gene. The relative expressions of genes were analyzed by the 2-ΔΔCT method. A combined ROC curve was made to predict the combined diagnostic capacity of the biomarkers.

**Table 2:** The primers’ list.

Primer Name	Sequence 5’ – 3’
F-GBP1	AACCATCAACCAGCAGGCTAT
R-GBP1	TTGTCCATCTGCTTCCAAGTC
F-HES4	CGCTCAGCTCAAAACCCTCATCC
R-HES4	AGGTGTCTCACGGTCATCTCCAG
F-IFI27	TCTGCAGTCACTGGGAGCAA
R-IFI27	CCCAATGGAGCCCAGGAT
F-LY6E	TGATGTGCTTCTCCTGCTTGAACC
R-LY6E	CCAAATGTCACGAGATTCCCAATGC
F-OTOF	GTGCTGGAGATGGAAGACCTTGAC
R-OTOF	CTGGCTTAGATCGCTTGTTGGAGAC
F-STAT1	AGCACCAGAGCCAATGGAACTTG
R-STAT1	GCAGGTTGTCTGTGGTCTGAAGTC
F-TTC21A	CCACATTCAGACTCCAGCCAGAC
R-TTC21A	CGCAGCCTCCATGTTAGCCTTC
F-XAF1	TTGATGTCAGAGCCCAAGCC
R-XAF1	AGCAGGATGCCACACTGAGA
F-ZCCHC2	GGCTCAGGTCCTTGTGGTTCTTG
R-ZCCHC2	CTCGGTACATTGGTCCAGGCATTG
F-GAPDH	TGACTTCAACAGCGACACCCA
R-GAPDH	CACCCTGTTGCTGTAGCCAAA

After the RT-qPCR validation, SLE biomarkers screened from the public database that we have previously published were compared, in order to further confirm the diagnostic value and specificity of the diagnostic markers we screened in SS patients.

### 2.6 Immune cell composition and immune cell analysis of diagnostic markers

The CIBERSORT algorithm was used for the immune cell analysis of the expression matrix of SS patients and healthy people. We edited an R language script to run CIBERSORT. CIBERSORT is used to estimate the cell composition of a single sample. The script reads the mixed gene expression data to be analyzed from the file system, normalizes and fits the mixed expression data using support vector machine, and then outputs the estimated relative content of each cell subset in the mixture after loading the necessary packages and functions. The script includes three functions: CoreAlg, doPerm, and CIBERSORT. The CoreAlg function defines the core algorithm, which accepts one cell type gene expression data and one mixed gene expression data, uses the support vector machine model to train and normalize the mixed data, and returns the relative content of each cell subset based on the model in the mixed cells. The main function is CIBERSORT, which accepts two file locations (unit-type gene expression data and mixed gene expression data), the number of permutations, and a Boolean value indicating whether quantization normalization is applied. The R package “vioplot” was adapted to visualize the ratios of 22 immune cells in SS and control groups (The ratio of cell represented the proportion of the number of a specific subtype immune cells in the blood cells of SS patients or healthy controls). The “corrplot” package was used to draw the heat map, which presented the quantitative correlation between different immune cells in SS patients. Additionally, the R package “ggplot2” was applied to investigate the relationship between the expression of the diagnostic markers and the ratios of immune cells using the Pearson method.

## 3 Results

### 3.1 PCA and DEGs screening

**Figure 2:**
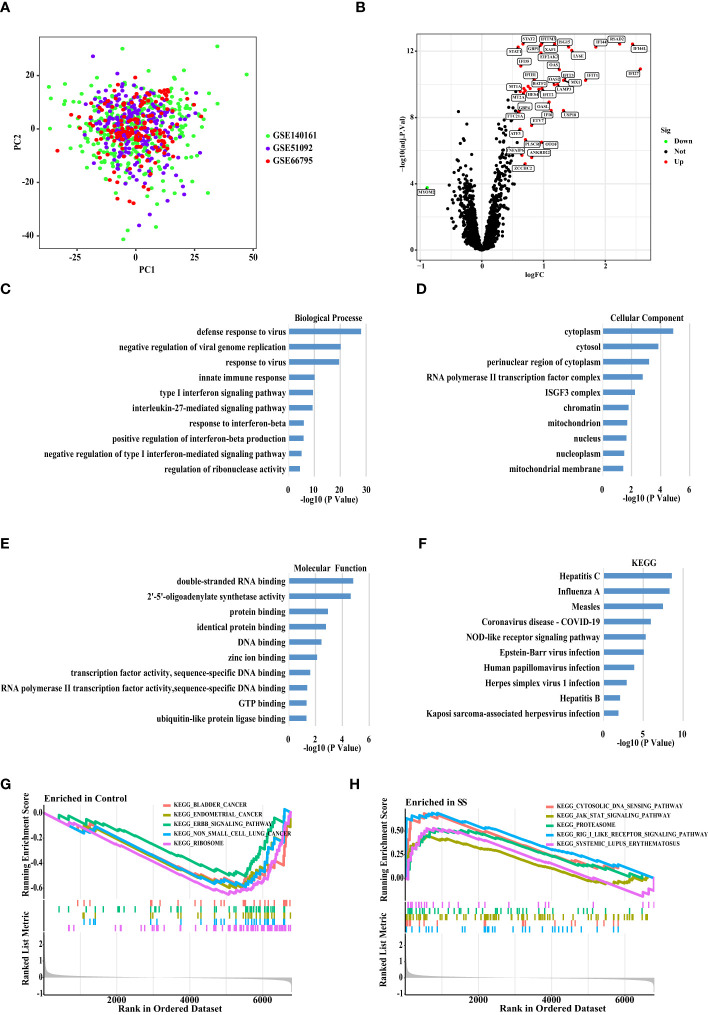
Principal Component Analysis and DEGs Screening between The SS versus Controls. **(A)** Principal component analysis (PCA) plot shows the distribution of each corrected sample. **(B)** Volcano map of DEGs between the whole blood of SS and control samples based on the discovery data set. P <0.0001 and Fold Change >1.5 considered significant. Green and red represented down-regulation and up-regulation, respectively (SS/Control). **(C)** The biological processes, **(D)** cellular components, and **(E)** molecular functions, **(F)** KEGG enrichment analysis of the DEGs. **(G, H)** The GSEA analysis of the SS and control samples.

After batch corrections using the R package “SVA”, we performed PCA on three datasets (GSE51092, GSE66795, and GSE140161) from GEO (**Figure 2A**). The result showed that each sample of the data set presented a uniform distribution, indicating that the normalization was performed appropriately. Then, we combined these three data sets into one data set (672 SS patients and 61 healthy people), and took about 80% of the samples from this data set as the discovery set. As a consequence, we obtained a total of 43 DEGs (**Figure 2B**) by comparing SS patients with healthy people (P < 0.0001, fold change > 1.5), and showed the expression of the DEGs in each healthy person and SS patient by a heat map (**Supplementary Figure S1**). Then, we discovered enrichment of response to type I interferon signaling pathway, innate immune response, and Epstein-Barr virus infection through the enrichment analysis of the DEGs (**Figures 2C–F**). Moreover, GSEA analysis revealed that pathways including cytoplasmic DNA sensing pathway, JAK STAT signaling pathway, proteasome, RIG-I-like receptor signaling pathway, and systemic lupus erythematosus were enriched in SS group compared to healthy group. Furthermore, compared with the healthy group, the SS group’s core genes are up-regulated (**Figures 2G, H**).

### 3.2 Identification and validation of diagnostic biomarkers for SS

**Figure 3:**
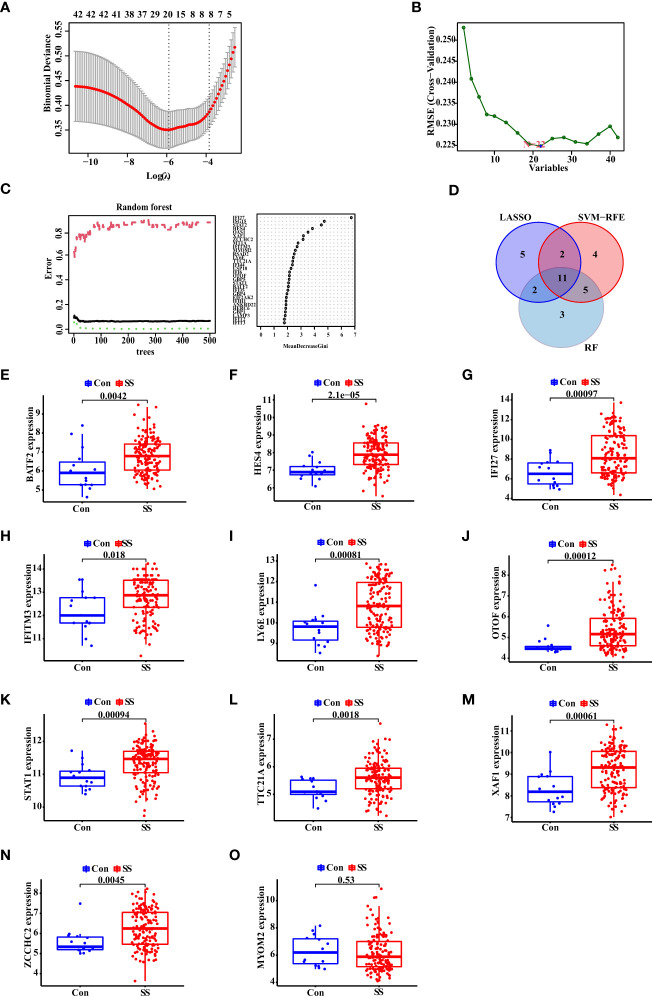
Identification and Validation of Diagnostic Biomarkers for SS. **(A)** The LASSO logistic regression, **(B)** SVM-RFE algorithm, and **(C)** RF algorithm of SS biomarker screening. **(D)** Venn diagram of the diagnostic biomarkers extracted by the three algorithms. **(E–O)** The expression of diagnostic biomarkers based on the validation data set. “Con” represented the control samples, and “SS” represented the SS patients.

To explore the biomarkers of SS, we applied the LASSO regression, the SVM-RFE algorithm and RF algorithm. As a result, 20 probable biomarkers were picked out by the LASSO regression algorithm, 22 by the SVM-RFE algorithm, and 21 by the RF algorithm (**Figures 3A–C**). Subsequently, we overlapped the 20, 22 and 21 probable biomarkers to obtain 11 genes, including BATF2, HES4, IFI27, IFITM3, LY6E, OTOF, STAT1, TTC21A, XAF1, ZCCHC2, and MYOM2 (**Figure 3D**). To verify the accuracy of the results, we conducted validation of the 11 genes using the verification data set. As seen in **Figures 3E–O**, the expression trend of the 11 genes was found to be consistent in both discovery and validation data sets, while MYOM2 had no statistical significance. On this account, we chose BATF2, HES4, IFI27, IFITM3, LY6E, OTOF, STAT1, TTC21A, XAF1, and ZCCHC2 for the following analysis.

### 3.3 The diagnostic capability of 12 biomarkers for SS

**Figure 4:**
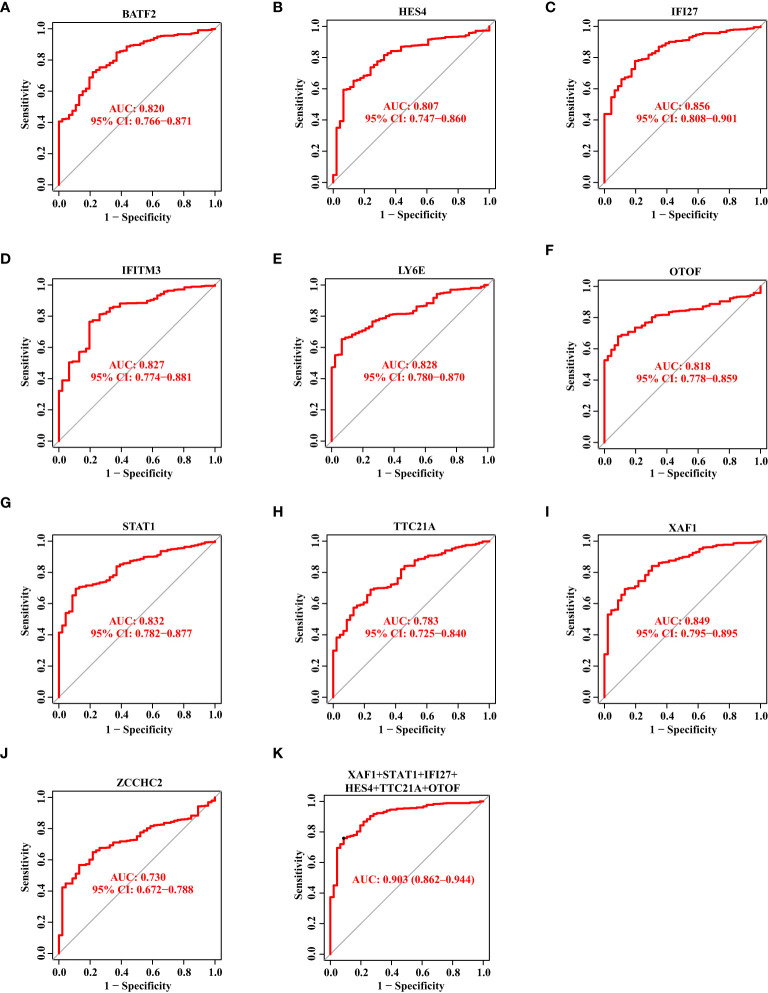
The Diagnostic Capability of Potential Biomarkers for SS Based on The Discovery Data Set. **(A–J)** The ROC curve of BATF2, HES4, IFI27, IFITM3, LY6E, OTOF, STAT1, TTC21A, XAF1, and ZCCHC2 based on the discovery data set. **(K)** The ROC curve showing the AUC value of XAF1, STAT1, IFI27, HES4, TTC21A, and OTOF based on the discovery data set.

Thereafter, we showed the ROC curves of 10 biomarkers, from which we could see the AUC values of BATF2, HES4, IFI27, IFITM3, LY6E, OTOF, STAT1, TTC21A, XAF1, and ZCCHC2 in the discovery data set. In **Figures 4A–J**, we observed these genes with the AUC value of 0.820, 0.807, 0.856, 0.827, 0.828, 0.818, 0.832, 0.783, 0.849, and 0.730, respectively. Then, we combined these genes into one signature and found that the combination of XAF1, STAT1, IFI27, HES4, TTC21A, and OTOF resulted in a relatively high AUC value of 0.903 (**Figure 4K**).

**Figure 5:**
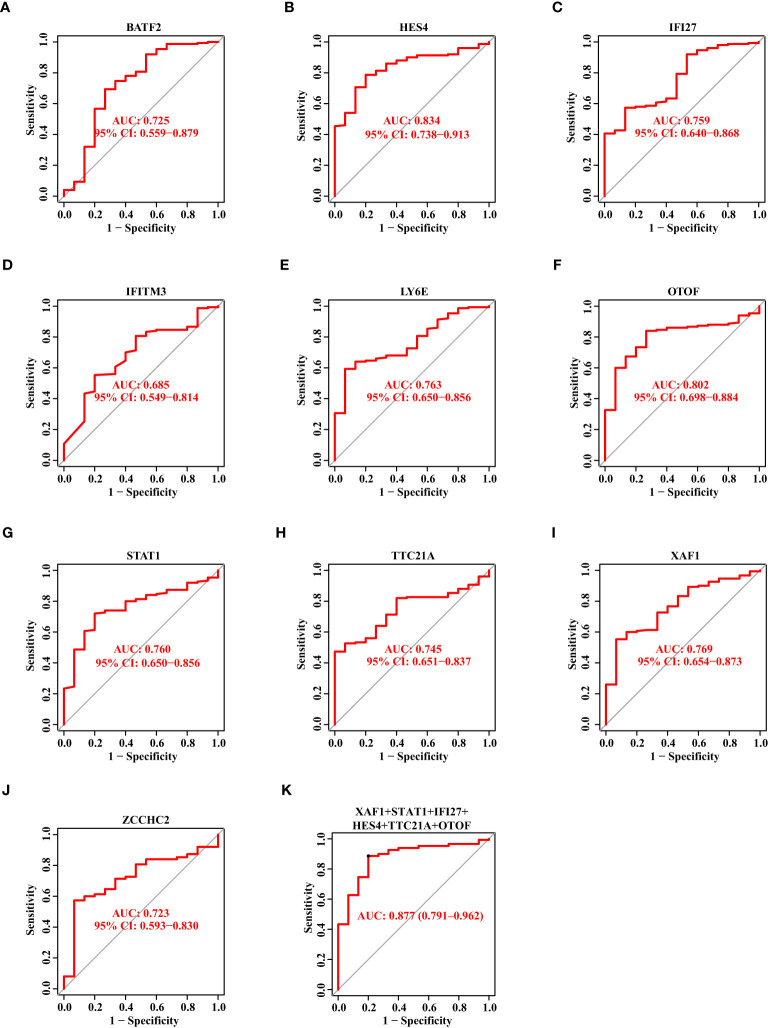
The Diagnostic Capability of Potential Biomarkers for SS Based on The Verification Data Set. **(A–J)** The ROC curve of BATF2, HES4, IFI27, IFITM3, LY6E, OTOF, STAT1, TTC21A, XAF1, and ZCCHC2 based on the verification data set. **(K)** The ROC curve showing the AUC value of XAF1, STAT1, IFI27, HES4, TTC21A, and OTOF based on the verification data set.

Likewise, we drew the ROC curves of 10 biomarkers based on the validation data set. The AUC value of BATF2, HES4, IFI27, IFITM3, LY6E, OTOF, STAT1, TTC21A, XAF1, and ZCCHC2 was 0.725, 0.834, 0.759, 0.685, 0.763, 0.802, 0.760, 0.745, 0.769, and 0.723, respectively (**Figures 5A–J**). When we combined XAF1, STAT1, IFI27, HES4, TTC21A, and OTOF as one signature, the AUC value reached 0.877 (**Figure 5K**).

### 3.4 The validation of the potential diagnostic markers using our own Chinese cohort

**Figure 6:**
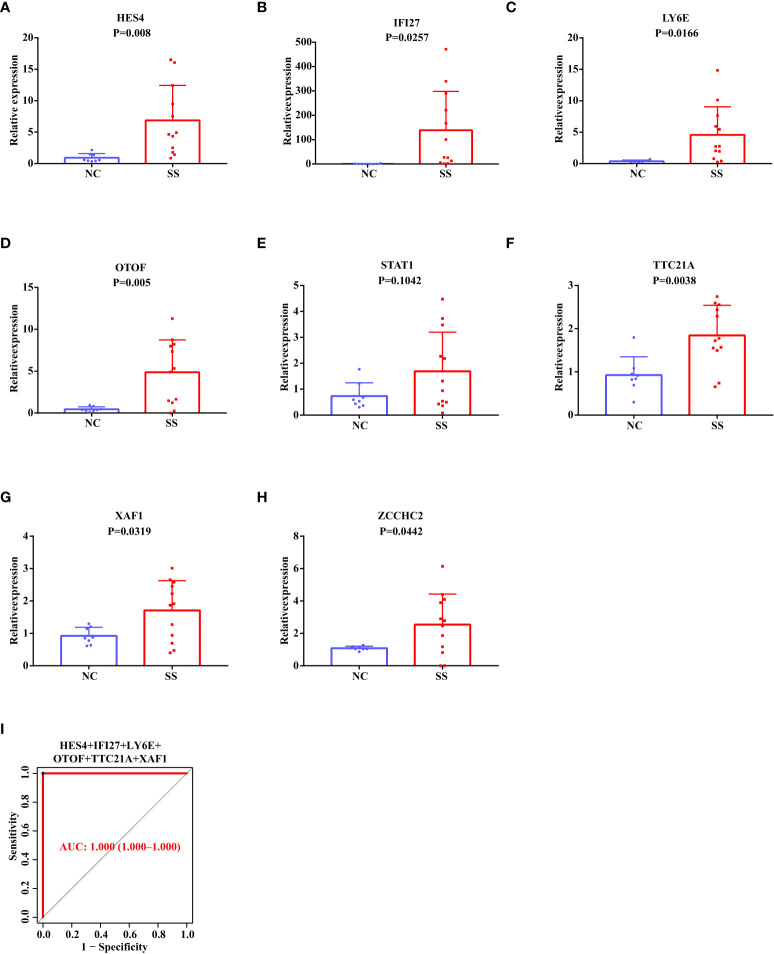
RT-qPCR Validation of The Potential Diagnostic Markers using Our Own Chinese Cohort. **(A–H)** The relative mRNA expression levels of HES4, IFI27, LY6E, OTOF, STAT1, TTC21A, XAF1, and ZCCHC2 in the PBMC of SS patients and healthy people. **(I)** The ROC curve showing the AUC value of HES4, IFI27, LY6E, OTOF, TTC21A, and XAF1 based on the Chinese cohort.

Based on the above results in the discovery data set and validation data set, we selected eight candidate biomarkers for RT-qPCR validation in 14 Chinese SS patients and 10 Chinese healthy controls (the result of the Ct values was shown in **Supplementary Table S1**). After analysis, we found that the mRNA expression levels of HES4, IFI27, LY6E, OTOF, STAT1, TTC21A, XAF1, and ZCCHC2 were consistently higher in SS patients than in healthy controls (**Figures 6A–H**). However, STAT1 did not show statistical significance. The AUC value of the combined ROC curve of HES4, IFI27, LY6E, OTOF, TTC21A, and XAF1 of the validation cohort reached 1 (**Figure 6I**), which showed an outstanding diagnostic effects to predict SS from HCs.

As a result, among the Chinese population, HES4, IFI27, LY6E, OTOF, TTC21A, XAF1, and ZCCHC2 are more likely to be SS biomarkers. Remarkably, HES4, OTOF, TTC21A, and ZCCHC2 are novel discovered biomarkers for SS that have not been reported in published articles.

In our previous article on SLE biomarkers (22), we downloaded the SLE gene expression matrix from the public database. After screening and validation, six markers (ABCB1, EIF2AK2, HERC6, ID3, IFI27, and PLSCR1) were obtained that had diagnostic value for SLE (the SLE biomarkers were shown **Supplementary Table S1**). By comparing with the seven biomarkers we screened in SS (HES4, IFI27, LY6E, OTOF, TTC21A, XAF1, and ZCCHC2), we found that IFI27 had diagnostic value in both diseases. Additionally, LY6E and XAF1 are also potential biomarkers in SLE reported by others (23, 24). As a result, HES4, OTOF, TTC21A, and ZCCHC2 were more likely to be considered as specific diagnostic biomarkers of SS.

### 3.5 The ratio changes of immune cells in SS Patients, and their correlation with the expression of diagnostic markers

**Figure 7:**
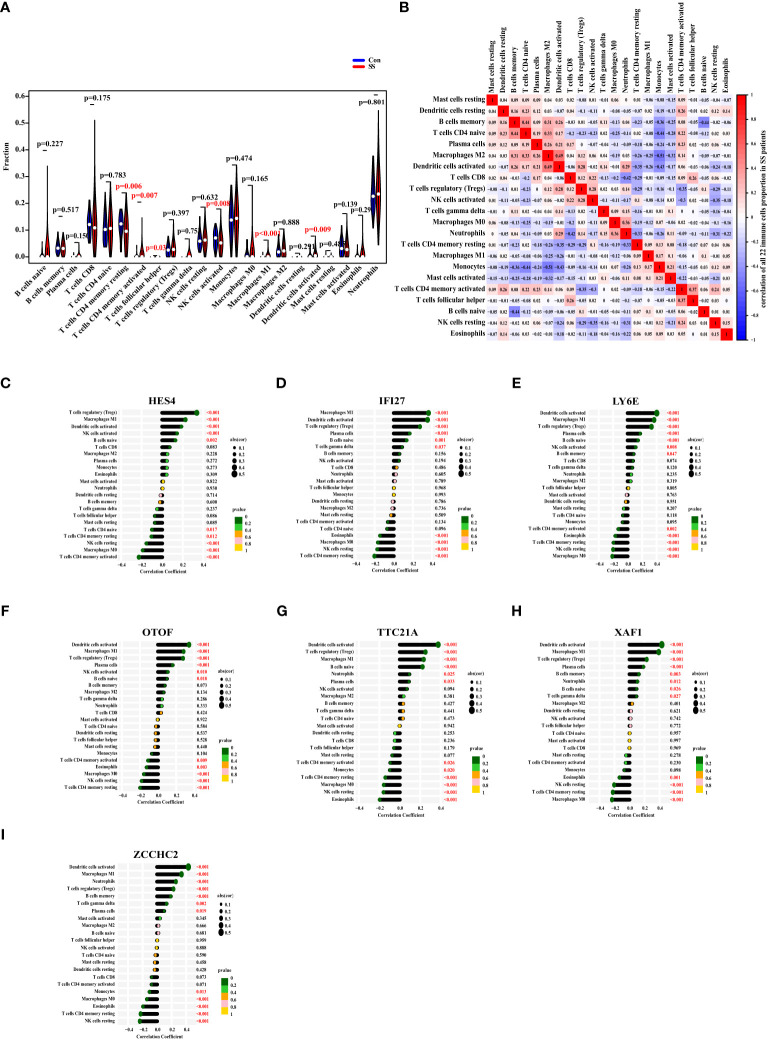
The Ratio Changes of Immune Cells in SS Patients, and Their Correlation with The Expression of Diagnostic Markers. **(A)** The fraction of 22 types of immune cells of SS patients (red) and healthy people (blue). **(B)** The heat map displaying the 22 different types of immune cells’ proportional association in SS patients. **(C–I)** The correlation between the expression of HES4, IFI27, LY6E, OTOF, TTC21A, XAF1, and ZCCHC2 with the levels of immune cells in SS. The size of the dots represented the correlation strength. The p value was represented by the color of the dots.

First, we used the CIBERSORT algorithm to examine the ratio of 22 immune cells in healthy controls and SS patients of the discovery data set. The finding revealed that the proportions of CD4 memory resting T cells and activated NK cells in SS were considerably lower than those in the control group, while CD4 memory active T cells, follicular helper T cells, macrophages M1, and activated dendritic cells were higher in SS (**Figure 7A**). Subsequently, we explored the relationships between the ratios of the 22 different immune cell types in SS patients. From the heat map, we found that the proportions of memory B cells and CD4 naïve T cells, the levels of activated dendritic cells and macrophages M2, had a moderate positive link, respectively. Besides, the ratio of neutrophils and CD8 T cells, the levels of memory B cells and naïve B cells, the degrees of monocytes and CD4 naïve T cells, macrophages M2, and activated dendritic cells had a negative link (**Figure 7B**).

Eventually, we investigated the correlation between the expression of HES4, IFI27, LY6E, OTOF, TTC21A, XAF1, and ZCCHC2 in SS patients and the ratio of immune cell. As a result, the expression of HES4 was positively correlated with the levels of regulatory T cells (Tregs), macrophages M1, activated dendritic cells, activated NK cells, and naïve B cells, and negatively correlated with the ratios of CD4 naive T cells, CD4 memory resting T cells, resting NK cells, macrophages M0, and CD4 memory activated T cells (**Figure 7C**). The expression of IFI27 was positively linked with the degrees of macrophages M1, activated dendritic cells, regulatory T cells, plasma cells, naive B cells, and gamma delta T cells, and negatively linked with eosinophils, macrophages M0, resting NK cells, and CD4 memory resting T cells (**Figure 7D**). The expression of LY6E was positively correlated with the ratios of activated dendritic cells, macrophages M1, Tregs, plasma cells, naive B cells, activated NK cells, and memory B cells, and negatively correlated with CD4 memory activated T cells, eosinophils, CD4 memory resting T cells, resting NK cells, and macrophages M0 (**Figure 7E**). The OTOF expression was positively correlated with the ratios of activated dendritic cells, macrophages M1, Tregs, plasma cells, activated NK cells, and naive B cells, and negatively correlated with CD4 memory activated T cells, eosinophils, macrophages M0, resting NK cells, CD4 memory resting T cells (**Figure 7F**). The expression of TTC21A was positively correlated with the ratios of activated dendritic cells, Tregs, macrophages M1, naive B cells, neutrophils, and plasma cells, and negatively correlated with CD4 memory activated T cells, monocytes, CD4 memory resting T cells, macrophages M0, resting NK cells, and eosinophils (**Figure 7G**). The expression of XAF1 was positively correlated with the ratios of activated dendritic cells, macrophages M1, Tregs, plasma cells, memory B cells, neutrophils, naïve B cells, and gamma delta T cells, and negatively correlated with eosinophils, resting NK cells, CD4 memory resting T cells, and macrophages M0 (**Figure 7H**). The expression of ZCCHC2 was positively correlated with the ratios of activated dendritic cells, macrophages M1, neutrophils, Tregs, memory B cells, gamma delta T cells, and plasma cells, and negatively correlated with monocytes, macrophages M0, eosinophils, CD4 memory resting T cells, and resting NK cells (**Figure 7I**).

On the whole, the expressions of HES4, IFI27, LY6E, OTOF, TTC21A, XAF1, and ZCCHC2 were most closely linked to the ratios of activated dendritic cells, macrophages M1, and Tregs.

## 4 Discussion

SS is a systemic chronic autoimmune disease with complex pathogenesis (25, 26). The diagnosis of SS relies on blood samples, an evaluation of lacrimal and salivary gland function, and labial salivary gland biopsies (26, 27), which is arduous and time-consuming with an invasive way. Therefore, trying to identify reliable and sensitive biomarkers can be used to diagnose SS.

In this study, we used three machine learning methods to screen potential diagnostic biomarkers of SS. We used the same expression matrix files (GSE51092, GSE66795, and GSE140161) to screen biomarkers and verify the screening markers. The results showed that the expression trends of biomarkers screened in the discovery data set were consistent with those in the validation data set, indicating that our screening strategy was reliable. In addition, HES4, IFI27, LY6E, OTOF, TTC21A, XAF1, and ZCCHC2 were found to be more suitable biomarkers for SS in the Chinese population after validation in our own Chinese cohort (n_SS_ =14, n_normal_ =10). However, a larger cohort may be required for further validation in future research. Due to the small sample size, there was a quite difference in age between SS and healthy control. The biomarkers we validated were somewhat impacted by the age mismatch. Previous studies have also shown that the levels of some indicators vary in different age groups. The elderly-onset of SS was associated with lower frequency of SS-related inflammatory arthritis, anti-Ro/SSA and anti-La/SSB positivity, and lower levels of RF, C3, and C4 (28). Hence, we hope to validate these potential biomarkers we screen in the future using larger cohorts with better cohort design.

Besides, the data set cohorts we downloaded from the GEO database did not provide information about ethnicity, age, disease duration, treatment, etc., which became a limitation in our study. In some extent, the absence of age and sex information may make it impossible to judge the generalization ability of markers. The biomarkers screened from patients of different age ranges or different sex ratios may be different. In addition, disease duration and treatment information may provide us with more useful hints.

Biomarkers for SS have been reported in some studies (17, 29, 30). In 2021, Li et al. revealed biomarkers of salivary glands in SS patients (31). Compared with this report, our research has the following expansion and in-depth research: First, we use three machine learning methods, which shows that our method has stronger recognition ability of biomarkers compared with the intersection method adopted by Li et al. Secondly, we use blood samples, which are easier to obtain than salivary glands, non-invasive to the patient and easier to repeat in later clinical monitoring. Finally, we have a larger sample size, which makes the markers we screen more reliable, accurate, and generic. Additionally, the innate immune response, type I interferon signaling pathway, and so on, which are common pathways connected to immunological illnesses, are included in the pathways we have enriched. This indicated that the biomarkers we investigated in DEGs could be potential biomarkers of SS for clinic. Furthermore, the biomarkers MS4A1, CD19, TCL1A, CCL19, CXCL9, CD3G, and CD3D that were ultimately screened from salivary glands did not overlap with the biomarkers that we screened from blood samples in this research, which may offer us a hint that the biomarkers from different tissues are noticeably different.

Disruption the balance of immunity cells has been observed in autoimmune diseases, such as multiple sclerosis (32), systemic lupus erythematosus (33, 34), rheumatoid arthritis (35). Biological abnormalities associated with B lymphocytes are a hallmark of SS (36–38). The tissue-resident Fc Receptor-Like 4 (FcRL4)+ B cell subset was recently reported to be a key driver in SS patients with mucosa-associated lymphoid tissue (MALT)-lymphomas, and FcRL4+ B cells are expanded in SS patients’ inflammatory tissues (39, 40). Apart from B cells, T cells are also important players in SS (41, 42). The normal balance of different subsets of CD4 +T cells, such as Th1, Th2, Th17, follicular helper T cells (Tfh), and Tregs, was found to be disrupted in SS patients (43, 44). It can be seen that immune system disorder played an important role in immune diseases. Thus, we explored the ratio changes of immune cells in SS patients, and investigated their correlation with the expression of diagnostic markers. Besides, we looked into the degree of correlation between Tfh and plasma blast using publicly available datasets, and we hypothesized that the lack of a positive result might be due to the fact that these studies used various inclusion criteria, sample processing techniques, data collection techniques, and data quality control methods.

Consistent with our results, Kimoto’s team has reported that IFI27 gene expression levels were considerably higher in the SS patients when compared to healthy controls (45). The interferon type I inducible genes LY6E and XAF1 were both increased in SS patients, and the two genes were found to be closely related and identified as the hub genes of SS (46, 47). HES4 has been reported to promote T cell development in the presence of Notch1 signaling (48). No previous study has reported that TTC21A is involved in development of SS. However, increased level of TTC21A expression was significantly associated with tumor status and lymph node status (49). There were few studies on the ZCCHC2 gene in the literature.

Interestingly, in our previous study of SLE based on the public database (22), we found that IFI27 is also a candidate marker for SLE. We concluded that IFI27 may be a biomarker for various immune diseases. In addition, we found that LY6E and XAF1 have also been reported as potential markers in SLE (23, 24). To the best of our knowledge, among the seven biomarkers of SS obtained in this study, HES4, OTOF, TTC21A, and ZCCHC2 can be used as specific diagnostic biomarkers of SS. Furthermore, in the **Supplementary Table S1**, we summarized the functions of these genes and their connections with SS or autoimmunity.

## 5 Conclusion

All in all, we identified seven genes (HES4, IFI27, LY6E, OTOF, TTC21A, XAF1, and ZCCHC2) as prospective SS biomarkers, which were more suitable for Chinese populations. In addition, we observed quantitative changes in six different types of immune cells in SS patients. Finally, we explored the relationship between the expression of the seven genes and the proportion of different immune cells. Our study provides potential biomarkers for Chinese SS patients and elucidates the relationship between gene expression and ratios of immune cells.

## Data availability statement

The datasets presented in this study can be found in online repositories. The names of the repository/repositories and accession number(s) can be found in the article/**Supplementary Material**.

## Ethics statement

The studies involving human participants were reviewed and approved by the Ethics Committee of Shenzhen People’s Hospital. The patients/participants provided their written informed consent to participate in this study.

## Author contributions

WZ conceived and designed the project. YZ performed the literature search and wrote the manuscript. SL provided data analysis support. XH and DL contributed to clinical sample collection. JL and LL performed the literature search. ZZ and WC performed the experiments. DT and YD supervised the study. All authors contributed to the article and approved the submitted version.
