# Supplementary figures and images for: Screening biomarkers for Sjogren’s Syndrome by computer analysis and evaluating the expression correlations with the levels of immune cells
Source: Front Immunol. 2023 Jun 13;14:1023248. doi: 10.3389/fimmu.2023.1023248 (PMC10294232; doi:10.3389/fimmu.2023.1023248)

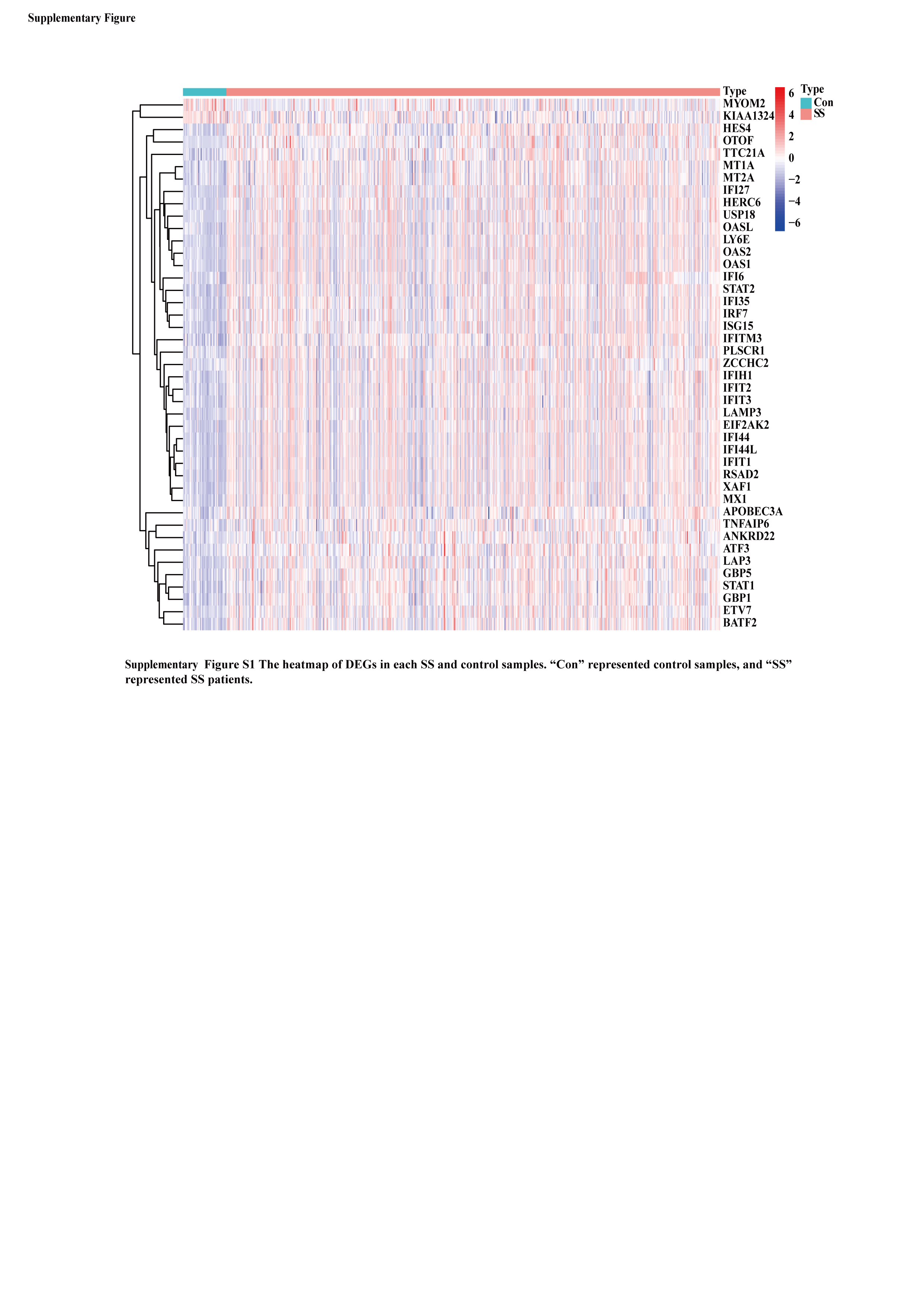

Supplement: Supplementary file 1 [file Image_1.tif]
